# The Utility of Pharmacogenetics Testing in Psychiatric Populations

**DOI:** 10.3390/jpm11121262

**Published:** 2021-12-01

**Authors:** Gesche Jürgens

**Affiliations:** Clinical Pharmacology Unit, Zealand University Hospital, DK-4000 Roskilde, Denmark; gju@regionsjaelland.dk

**Keywords:** pharmacogenetic testing, psychiatric disorders, pharmacotherapy

## Abstract

The implementation of pharmacogenetic tests including multiple gene variants has shown promising potential as a decision-making tool for optimizing psychopharmacological treatment regimens and reducing treatment costs. However, the varying clinical validity of gene variants included in pharmacogenetic test batteries, and inconsistencies in their translation into medical recommendations between commercially available pharmacogenetic tests, complicates their rational implementation. Thus, there is a need for well-designed, reproducible studies documenting the clinical significance of the various genetic variants.

Callegari and colleagues [1] show in their study a significant financial saving associated with an adaptation of the psychopharmacological treatment to a pharmacological test battery consisting of a combination of both pharmacokinetic and pharmacodynamic markers, which translate into an easily understandable risk stratification for drugs according to a traffic light principle. The cost estimate is calculated over an entire year before and after pharmacogenetic testing. The savings go hand in hand with a significant reduction in hospitalization days, as well as fewer inquiries to the emergency services, which are clinically relevant outcome parameters. Although relatively small and neither blinded nor randomized, this study contributes as an important piece in the big picture of the utility and implementation of pharmacogenetics in our clinical everyday life. However, we should not be blind to the fact that there is still much to understand.

In recent years, there has been a trend towards the combination of several gene variants rather than using them individually. This makes good sense on a theoretical level as several potentially competing genetic variants relevant to the current pharmacological treatment can be detected simultaneously. However, it also involves combining gene variants with different clinical validity, some of them without broader consensus on how the result should be translated into a medical decision. For example, SNP G143E (rs71647871) is the only Carboxyesterase 1 (*CES1*) variant identified to date that would be expected to alter the therapeutic results of CES1 substrate drugs, such as methylphenidate [2]. However, *CES1* gene variation only partially explains the pharmacokinetic variability [3]. A better understanding of the regulation of *CES1* expression and activity would be desirable before this gene variant was included in a pharmacogenetic test battery. Another example is the *CYP2C19* haplotype *1F, which is associated with increased metabolism, but apparently only becomes phenotypically significant in the presence of additional inducers [4]. Its’ clinical significance as a tool for the dose optimization of psychiatric substrate drugs such as clozapine is still unclear [5,6]. An over-interpretation of the clinical significance of these gene variants may deny patients a potentially effective treatment on a vague scientific basis. Not to mention, that the majority of gene variants have only been studied in relation to a single drug treatment. Despite this, polypharmacy is commonly used for the treatment of psychiatric disorders [7].

In addition, commercialization seems to have overtaken the scientific validation process of these tests, and testing by private providers, and possibly even on the patient’s own initiative, may challenge clinicians to incorporate test results in their prescription routines without sufficient knowledge of their interpretation and limitations. This is further complicated by the fact that different providers of commercial pharmacogenetic tests translate test results into different medical treatment recommendations [8].

Despite these concerns, there is an accumulating amount of evidence associating pharmacogenetic testing with better treatment outcomes in some psychiatric populations. Thus, Rosenblat and colleagues show in their meta-analysis from 2017 that the use of these tests is associated with both improved response and remission rates in patients with major depression disorders, although the authors point out significant methodological limitations [9]. A review of economic savings associated with pharmacogenetic testing in this patient group, conducted by the same author group, showed no clear effect [10]. In other therapeutic areas, the evidence is more sparse. Thus, a recently published RCT examining the effect of *CYP2D6* and *CYP2C19* genotyping in a population of patients with schizophrenia, shows no effect on persistence, treatment effect, or the side effects of the antipsychotic treatment [11]. Interestingly, however, an economic analysis based on data from this RCT shows that the additional costs associated with the slow and rapid metabolism of CYP2D6 and CYP2C19 is saved by routine use of pharmacogenetic testing [12].

The rational implementation of pharmacogenetic tests in practice has shown promising potential as a decision-making tool for optimizing psychopharmacological treatment regimens and reducing treatment costs. However, it is important that the implementation of these tests is based on replicable scientific evidence and that we fully understand the underlying mechanisms. Equally important is that the recommendations derived from pharmacogenetic tests are based on a broad consensus of recognized professional bodies such as the CPIC (Clinical Pharmacogenetics Implementation Consortium) and DPWG (The Dutch Pharmacogenetics Working Group). Batteries of pharmacogenetic tests should not be implemented in clinical practice as a kind of black box test where it is unknown whether the effect obtained is actually due to the pharmacological treatment adapting the pharmacogenetic test result, or is confounded by the doctor, for example, giving the patient’s pharmacological treatment greater attention.

## Data Availability

Not applicable.
